# Pulsed Electric Fields as an Effective Tool for *Toxoplasma gondii* Inactivation

**DOI:** 10.3390/foods15081447

**Published:** 2026-04-21

**Authors:** Vanesa Abad, Daniel Berdejo, Juan Manuel Martínez, Nabil Halaihel, João Luis Garcia, Ignacio Álvarez-Lanzarote, Susana Bayarri, Guillermo Cebrián

**Affiliations:** 1 Departamento de Producción Animal y Ciencia de los Alimentos, Tecnología de los Alimentos, Facultad de Veterinaria, Instituto Agroalimentario de Aragón, IA2 (Universidad de Zaragoza-CITA), 50013 Zaragoza, Spain; vabad@unizar.es (V.A.); j.martinez@unizar.es (J.M.M.); ialvalan@unizar.es (I.Á.-L.); guiceb@unizar.es (G.C.); 2 Departamento de Producción Animal y Ciencia de los Alimentos, Nutrición y Bromatología, Facultad de Veterinaria, Instituto Agroalimentario de Aragón, IA2 (Universidad de Zaragoza-CITA), 50013 Zaragoza, Spain; berdejo@unizar.es; 3 Departamento de Patología Animal, Sanidad Animal, Facultad de Veterinaria, Universidad de Zaragoza, 50013 Zaragoza, Spain; nhk@unizar.es; 4 Department of Preventive Veterinary Medicine, Protozoology Laboratory, Universidade Estadual de Londrina—UEL, P.O. Box 6001, Londrina 86050-970, PR, Brazil; jlgarcia@uel.br

**Keywords:** parasite, oocysts, bradyzoites, water, meat, food safety, non-thermal processing

## Abstract

*Toxoplasma gondii* is an intracellular protozoan transmitted via environmentally resistant oocysts present in food and water, as well as through the consumption of meat containing infective bradyzoites. This study evaluated the inactivation of *T. gondii* oocysts and bradyzoites (ME-49 strain) by Pulsed Electric Field technology (PEF). Treatment efficacy was determined by mouse bioassay combining brain qPCR and indirect immunofluorescence (IFA), with complementary qPCR in Hs27 cells. The infectious dose (ID_50_) of *T. gondii* was estimated at 34.6 oocysts. PEF-treated oocysts (15 kV/cm; 50 kJ/kg; 225 µs) showed a significant reduction in infectivity compared with untreated controls; accordingly, the dose required to establish infection increased to 85.3 oocysts after PEF treatment. Brain qPCR and IFA were highly correlated, whereas heart tissue was less sensitive. Bradyzoites recovered from PEF-treated meat (3.3 kV/cm; 27 kJ/kg; 1600 µs) showed a 50% infectivity reduction compared with untreated samples. In vitro assays confirmed an in vivo reduction in infectivity, indicating that cell cultures can serve as an ethical and efficient tool for preliminary viability assessment. This is the first evidence of *T. gondii* inactivation by PEF, highlighting its potential as a non-thermal strategy. Further studies are needed to optimize treatment parameters.

## 1. Introduction

*Toxoplasma gondii* is an obligate intracellular protozoan parasite belonging to the phylum *Apicomplexa*. Disseminated worldwide, it has a broad host range, infecting nearly all warm-blooded animals, including humans [1]. *T. gondii* has a heteroxenous life cycle characterized by sexual reproduction in definitive hosts (members of the *Felidae* family) and asexual multiplication in a wide range of intermediate hosts [1]. In felids, sexual development occurs in the intestinal epithelium, resulting in the shedding of unsporulated oocysts through feces.

Once excreted into the environment, oocysts require 1 to 5 days to sporulate under optimal conditions [1,2,3]. Sporulated oocysts are protected by a complex multilayered structure, which provides remarkable resistance to environmental stresses [4,5]. Each sporulated oocyst contains two sporocysts, each with four sporozoites capable of initiating infection in intermediate hosts, such as birds, rodents, livestock, and humans [6]. When ingested by intermediate hosts, sporozoites are released in the intestine and invade intestinal epithelial cells, where they differentiate into tachyzoites that disseminate systemically, infecting various tissues and subsequently differentiating into bradyzoites that form long-lived tissue cysts primarily in the brain, muscle, and heart [7,8,9].

As intermediate hosts, humans typically become infected through ingestion of either sporulated *T. gondii* oocysts present in contaminated water, fruits, and vegetables that have come in contact with feces from infected felids or raw/undercooked meat containing tissue cysts with bradyzoites [10,11]. The infection poses significant risks during pregnancy, as primary maternal infection can result in transplacental transmission of the parasite to the fetus. This congenital infection is associated with severe outcomes such as miscarriage, stillbirth, or neurological and ocular damage in the newborn [12]. Immunocompromised individuals are also susceptible to reactivation of latent infection or severe primary disease, often manifested as neurological symptoms and brain lesions due to tachyzoite proliferation [13].

Due to their high environmental resistance and role in disease transmission, effective inactivation methods for *T. gondii* oocysts are essential. Heat treatment has been shown to render oocysts non-infective at 55 °C for 2 min or 60 °C for 1 min, whereas freezing at −21 °C for 28 days is insufficient for inactivation. Ultraviolet radiation, including pulsed and continuous UV-C, is effective in water at doses exceeding 1000 mJ/cm^2^. High hydrostatic pressure processing also represents a viable alternative, with effective treatments reported at 340 MPa for 1 min. In contrast, chemical treatments show variable efficacy: chlorination at 100 mg/L for 24 h is ineffective, while ammonia at 5.5% requires prolonged exposure (3 h) to achieve inactivation [14,15,16,17,18,19]. Furthermore, additional treatment of meat to eliminate viable *T. gondii* tissue cysts would provide a means to protect consumers. Meat-processing techniques, such as heat treatment, freezing, high-hydrostatic-pressure processing (HPP), and irradiation, have demonstrated parasite-inactivating potential, although meat quality properties could slightly be compromised depending on the technology [20,21,22,23,24,25,26].

Overall, these limitations highlight the need for other (non-thermal) technologies capable of ensuring parasite inactivation while preserving food quality. Pulsed Electric Field (PEF) treatments consist of subjecting a product, placed between two electrodes, to high-intensity electric fields (between 0.5 and 30 kV/cm) by applying intermittent pulses of short duration (of the order of microseconds) without increasing the product’s temperature [27]. This technology has shown promising results in inactivating bacteria and some other parasites [27,28,29]. Thus, Martinez et al. [29] highlight the application of PEF in water sanitation, reporting successful inactivation of parasites such as Giardia, Cryptosporidium, and Ascaris, among others, but also report that the application of PEF for the inactivation of foodborne parasites in food products remains limited, with only two studies having evaluated PEF for the inactivation of *Anisakis* spp. in fish, demonstrating its potential as an alternative to conventional treatments [30,31]. Even though additional studies have been published more recently that further support the effectiveness of PEF for the inactivation of *Anisakis* in fish [32,33,34,35,36], its capability for inactivating *T. gondii* either in water (as oocysts) or in meat (as bradyzoites) has not yet been experimentally tested.

In light of the above, this non-thermal technology could offer an effective alternative to traditional treatments, including freezing and heat. Therefore, the objective of our study was to explore the effect of PEF treatments on the viability of *T. gondii* oocysts and bradyzoites. To assess treatment efficacy, cell culture assays and in vivo experiments on mice were conducted to determine oocyst infectivity after PEF treatments.

## 2. Materials and Methods

### 2.1. T. gondii Oocysts

Oocysts of *T. gondii* (strain ME-49) were obtained from cat feces (State University of Londrina, Brazil). The oocysts were stored at refrigeration temperatures (4 ± 1 °C) in 2% sulfuric acid until used (within 6 months of production).

Prior to PEF treatment, the oocyst suspension was filtered through a 200 µm pore size gauze to remove coarse material. The filtrate was then centrifuged at 1000 rpm for 60 s using an Eppendorf Centrifuge (MiniSpin Plus, Madrid, Spain). The supernatant was discarded, and the pellet was washed three times with Phosphate-Buffered Saline (PBS) (Sigma-Aldrich, Steinheim, Germany) of 1 mS/cm electrical conductivity, under the same centrifugation conditions. Following the washing steps, oocyst concentration was measured using a Thoma counting chamber and a Nikon microscope equipped with phase-contrast optics and an epifluorescence unit (Eclipse E400, Nikon, Tokyo, Japan) [37].

### 2.2. Bradyzoite-Infected Tissues

Experimental infection of mice with *Toxoplasma gondii* was carried out at the Centro de Investigación Biomédica de Aragón (CIBA) in Zaragoza (Spain). Swiss CD1 mice (Janvier Labs, Le Genest-Saint-Isle, France) were orally inoculated after one week of acclimatization with 250 µL of a solution containing 250 ME-49 strain oocysts as described in Section 2.4.1. After 60 days post-inoculation, mice were euthanized, eviscerated and deboned, and the skeletal muscle was pooled and stored under refrigeration until further use. Only the muscle from those mice that tested positive for *Toxoplasma gondii* infection according to the methodology described in Section 2.4.3 was used for subsequent assays.

All animal procedures, including inoculation, care, and euthanasia of mice, were carried out in compliance with the International Guiding Principles for Biomedical Research Involving Animals [38]. This project was authorized by the Biosecurity Commission (Project 11/22) and the Ethical Advisory Committee for Animal Experimentation at the University of Zaragoza (PI29_22).

### 2.3. PEF Treatment

#### 2.3.1. PEF Treatment of Oocysts

Oocysts of *T. gondii* in buffer solution of 1 mS/cm (concentration of 1.5 × 10^4^ oocysts/mL) were subjected to a PEF treatment of 15 kV/cm for 225 µs (50 kJ/kg), applying pulses of 3 µs at 1 Hz. The solution’s electrical conductivity was measured with a conductivity probe (Almemo FYA641LF series, Alhborn, Germany). The treatment chamber used consists of two parallel stainless-steel electrodes with a radius of 0.75 cm and an interelectrode distance (gap) of 0.25 cm.

The treatments were applied with a ScandiNova 6 MW apparatus (Modulator PG, ScandiNova, Uppsala, Sweden). The system applies square wave pulses with a fixed width of 3 µs and a frequency range of 0.5 to 300 Hz. Maximum voltage is 30 kV, and the current is 200 A.

#### 2.3.2. PEF Treatment of *T. gondii*-Infected Meat

Infected mouse tissue (meat from positive mice to *T. gondii*) was subjected to a PEF treatment of 3.3 kV/cm for 1600 µs (27 kJ/kg), applying pulses of 20 µs at 2 Hz, using a treatment chamber consisting of two parallel stainless-steel electrodes of 5 × 3 cm and an interelectrode distance (gap) of 3 cm. Mouse tissue (approx. 5 g) was wrapped in a gauze and immersed in a saline solution with an electrical conductivity of 1.5 mS/cm. The PEF equipment used for this part of the study was an EPULSUS-PM-10, 2 kW system from Energy Pulse System (Lisbon, Portugal). It applies square wave pulses of a maximum frequency of 200 Hz and pulse widths ranging from 1 to 200 µs. The maximum applicable voltage and current are 10 kV and 180 A, respectively.

Processing parameters were monitored with an oscilloscope (Tektronix, TDS 220, Wilsonville, OR, USA) connected to a high-voltage probe (Tektronix, P6015A, Wilsonville, OR, USA) and a current probe (Stangenes Industries Inc., Palo Alto, CA, USA). The temperature was kept under 35 °C in all experiments. The temperature of the treatment medium was measured as previously described [39].

The selected PEF treatments were chosen based on the structural characteristics and sizes of the targeted parasite. Previous studies have shown that electric field strengths between 10 and 20 kV/cm are typically required for effective microbial inactivation [39]. Considering *T. gondii* oocyst (10–12 µm) and bradyzoite (2–6 µm) sizes [40,41], the treatment applied to oocysts (15 kV/cm) was selected within the range commonly used for bacterial inactivation. In contrast, for bradyzoites present in meat, a lower electric field strength (3.3 kV/cm) was applied due to limitations of the treatment chamber and PEF system.

The efficacy of the PEF treatment was assessed using two methods: a mouse bioassay and a cell culture assay (see below).

### 2.4. Evaluation of PEF Treatment Efficacy by Mouse Bioassay

As previously stated, all procedures were authorized by the University of Zaragoza’s Biosecurity Commission (Project 11/22) and the Ethics Advisory Commission for Animal Experimentation (PI29_22), and were consistent with international principles for animal research (Directive 2010/63/EU).

The sample size was determined based on the scientific literature and previous studies performed in our laboratory. For each inoculation dose (see below) and treatment condition (PEF-treated or untreated), 4 mice (2 males and 2 females) were used, together with 4 negative controls (2 males and 2 females). This experimental design was repeated in 2 independent replicates. Investigators were blinded to treatment allocation during animal handling and outcome assessment.

#### 2.4.1. Evaluation of Oocyst Viability

Mice were orally inoculated with 250 µL of a solution containing varying doses (30, 60, 125, and 250) of PEF-treated or untreated (control) oocysts. Additionally, 4 mice were inoculated with a negative control consisting of 250 oocysts that had been inactivated by a heat treatment at 100 °C for 15 min. Groups consisting of two male and two female mice (25 g) were used to assess the viability of oocysts for each dose. Two independent replicates of each experiment were conducted.

#### 2.4.2. Evaluation of Bradyzoite Viability

Pepsin digestion of ≈30 g of infected tissue (treated and non-PEF-treated muscle samples) was performed following the protocol described by Dubey [16] with modifications by Bayarri et al. [42]. Three doses of the digestion extract were evaluated in batches of 4 mice: the undiluted digest, and 1/2 and 1/4 dilutions in PBS (Sigma-Aldrich, Steinheim, Germany). A 0.5 mL aliquot of the digestion extract was inoculated intraperitoneally into each mouse. Additionally, a batch of 4 mice was inoculated with a negative control consisting of a digestion extract from meat exposed to a heat treatment at 100 °C for 15 min.

#### 2.4.3. qPCR Detection of *T. gondii*

Two months after oral (oocysts) or intraperitoneal (bradyzoites) inoculation, mice were euthanized using CO_2_. The brain and heart were collected from each animal for subsequent analysis by quantitative polymerase chain reaction (qPCR). The same procedure as that described by Gracia et al. [26] was followed with minor modifications. Thirty milligrams of mouse brain or heart were homogenized in 300 µL of lysis buffer (Promega, Madison, WI, USA) and 37 µL of Proteinase K (Promega, Madison, WI, USA) using plastic hand-pestle rotating plungers in an Eppendorf, and subsequently incubated at 70 °C for 30 min. DNA extraction was performed using a Maxwell 16 Lev Blood DNA Kit (Promega, Madison, WI, USA), according to the manufacturer’s instructions. Amplification and detection of *T. gondii* were performed using a CFX Connect real-time PCR system (Bio-Rad Laboratories, Hercules, CA, USA) with GoTaq SYBR Green Master Mix (catalogue # A6002, Promega) and the primers listed in Table 1. The total reaction volume was 22 µL, consisting of 10 µL of nuclease-free water, 10 µL of master mix containing Taq polymerase, dNTPs, and buffer, 1.5 µL of sample, and 0.5 µL of SYBR Green for SYBR. The thermal cycling programme used for the qPCR procedure was as follows: an initial enzyme activation step at 95 °C for 3 min, followed by 40 cycles consisting of denaturation at 95 °C for 3 s, annealing at 55 °C for 20 s, and extension at 79 °C for 3 s. A melt curve analysis was performed at the end of the run, increasing the temperature from 60 to 95 °C in 0.5 °C increments to assess product specificity.

#### 2.4.4. Indirect Immunofluorescence Assay

One month after oral or intraperitoneal inoculation, blood samples were collected from the mice for an indirect immunofluorescence assay (IFA) (MegaFLUO TOXOPLASMA gondii kit; Eurovet Animal Health B.V., Bladel, The Netherlands) to detect antibodies against *T. gondii*. The serum dilutions we tested were 1:20, 1:40, 1:80, 1:160, 1:320, 1:640, 1:1280, 1:2560, and 1:5120. Positive and negative controls were likewise included. Slides were examined under a Nikon microscope equipped with an epifluorescence unit (Eclipse E400, Nikon, Tokyo, Japan).

### 2.5. In Vitro Evaluation of PEF Treatment Efficacy by Cell Culture Assays

For the cell culture assays, 24-well plates were seeded on day 0 with 10^4^ Hs27 cells per well (derived from *Homo sapiens* foreskin) (ATCC^®^, CRL-1634™, Manassas, VA, USA) and maintained in Dulbecco’s Modified Eagle’s Medium (DMEM) (VWR, Radnor, PA, USA) supplemented with 10% fetal bovine serum (Thermo Fisher Scientific, Waltham, MA, USA), 1% MEM Non-essential Amino Acid Solution (100×) (Sigma-Aldrich, Steinheim, Germany), 1% Penicillin–Streptomycin (10,000 units penicillin and 10 mg streptomycin per mL in 0.9% NaCl) (Sigma-Aldrich, Steinheim, Germany), and 0.1% Amphotericin B (250 μg/mL solution) (Sigma-Aldrich). Every two days, the medium was removed, the cells were washed with 500 μL of Dulbecco’s Phosphate-Buffered Saline, and 1 mL of fresh medium (DMEM) was added. Cells were incubated at 37 °C with 5% CO_2_, in accordance with the supplier’s instructions. Under these conditions, 100% confluence was reached after seven days.

#### 2.5.1. Evaluation of Oocyst Viability

Once confluence was achieved, each well was inoculated with PEF-treated or untreated (control) oocysts at one of the following doses: 4000, 400, 40, or 4 oocysts per well. The oocysts were suspended in the same DMEM-based culture medium described above, with a final volume of 1 mL per well. A negative control, consisting of 4000 oocysts exposed to 100 °C for 15 min to ensure complete inactivation, was also included. These samples were then incubated at 37 °C with 5% CO_2_ for seven days, with the medium replaced every two days.

Prior to the inoculation of oocysts into cell cultures, an excystation process was necessary. The latter was performed following the protocol described by Villegas et al. [6]. One millilitre of oocysts (control and PEF), at a concentration of 1.5 × 10^4^ oocysts/mL in PBS, was placed into a screw-cap microcentrifuge tube containing 0.5 g of 0.5 mm glass beads (BioSpec products, Bartlesville, OK, USA) and shaken in a BeadBeater (BioSpec products, Bartlesville, OK, USA) for 20 s at 250 rpm. The sample was then transferred to a new tube containing 500 µL of 10% bovine bile (Sigma-Aldrich, Steinheim, Germany) and incubated for 1.5 h at 37 °C. Subsequently, it was centrifuged at 17,000× *g* (Centrifuge 5418 R, Eppendorf) for 10 min at 4 °C. The supernatant was removed, and the pellet was resuspended in 1 mL of PBS, followed by a second centrifugation under the same conditions. After discarding the supernatant, the pellet was resuspended in 1 mL of supplemented DMEM.

#### 2.5.2. Evaluation of Bradyzoite Viability

Each well was inoculated with 1 mL of a 1/10 dilution in DMEM of the digested (see Section 2.4.2) PEF-treated or untreated meat. A negative control, consisting of 1 mL of a 1/10 dilution of heat-treated (100 °C for 15 min) digested meat, was also included. These samples were then incubated at 37 °C with 5% CO_2_ for seven days, with the medium replaced every two days.

#### 2.5.3. qPCR Detection of *T. gondii* in Cell Cultures

Seven days after inoculation in the cell culture, the well was processed to assess infectivity. The medium was removed, and 300 µL of lysis buffer (Promega, Madison, WI, USA), along with 50 µL of Proteinase K (Promega, Madison, WI, USA), was added. The plate was incubated at 50 °C for 2 h. An inoculation loop was used to thoroughly scrape the well surface, ensuring complete recovery of cellular content, which was subsequently transferred to a microcentrifuge tube. DNA extraction and detection by qPCR were then performed according to the protocol described in Section 2.3.1.

### 2.6. Infectivity Criteria, Model Fitting, and Statistical Analysis

The results of both mouse bioassay and cell culture were expressed as infectivity (Ct ≤ 38; IFA titer ≥ 1:20) or no infectivity (Ct > 38; IFA titer < 1:20) of *T. gondii* in tissue (brain/heart) or cell cultures.

Simple logistic regression was performed to fit the data using GraphPad PRISM^®^ software (GraphPadPrism version 8.00 for Windows, GraphPad Software, San Diego, CA, USA). For this purpose, when infectivity was confirmed, it was registered as “1”, and “0” if it was not.

All experiments were carried out at least in duplicate. Statistical analyses (Student’s *t*-test, ANOVA, and Tukey tests; Pearson and Spearman correlation tests, *p*-value < 0.05) were calculated using GraphPad PRISM^®^ statistical software.

## 3. Results

The effect of PEF treatments on *T. gondii* oocyst and bradyzoite viability, using two experimental models (mouse bioassay and cell culture), is described below.

### 3.1. Effect of PEF on T. gondii Oocysts

#### 3.1.1. Mouse Bioassay

##### qPCR of Mouse Brain

**Figure 1:**
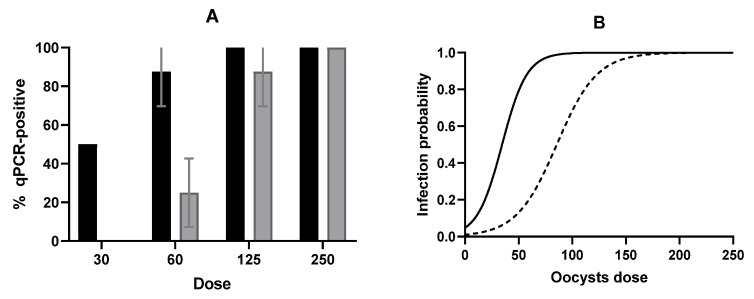
(**A**) Influence of *T. gondii* oocyst dose on the percentage of infected mice determined by qPCR of brain tissue samples. Control (untreated) samples (black bars) and PEF-treated samples (grey bars) (15 kV/cm, 50 kJ/kg, 225 µs). (**B**) Probability of infection in mice as a function of dose of control oocysts of *T. gondii* (continuous line) and PEF-treated oocysts of *T. gondii* (dashed line) (15 kV/cm; 50 kJ/kg; 225 μs). Error bars correspond to the standard deviation.

Figure 1 shows the percentage of mice infected after the oral administration of varying doses of PEF-treated and non-treated (control) oocysts as determined by qPCR of brain tissue (1A). At the highest dose tested (250 oocysts), all animals were qPCR-positive/infected. Decreasing the dose resulted in a progressive reduction in the percentage of animals infected, and the difference was more pronounced in the case of PEF-treated oocysts. Thus, when inoculated with 125 oocysts, one out of eight mice inoculated with PEF-treated oocysts already tested negative, whereas all the animals inoculated with non-treated oocysts were qPCR-positive. The difference between PEF-treated and control oocysts was even more pronounced at lower doses (25 vs. 87.5% and zero vs. 50%, for doses of 60 and 30 oocysts, respectively). All negative controls tested negative.

A simple logistic regression model based on qPCR data was developed to estimate the probability of infection following ingestion of control or PEF-treated oocysts at varying doses within the study range (Figure 1B). The model yielded an Area Under the Curve (AUC) of 0.9490 for the control group (*p* = 0.0225) and 0.9410 for the PEF-treated group (*p* = 0.0046). Based on this model, the 50% infectious dose (ID_50_) could be estimated, resulting in 34.57 ± 8.66 (95% CI = 12–51.21) oocysts for control oocysts. This dose was similar to that reported by Ware et al. [44], which describes an ID_50_ of 24 oocysts. In the case of oocysts subjected to a PEF treatment of 15 kV/cm for 225 µs (50 kJ/kg), the ID_50_ was 85.33 ± 12.61 (95% CI = 62.05–113.8) oocysts, reflecting a marked reduction in infectivity following PEF treatment.

##### qPCR of Mouse Heart

**Figure 2:**
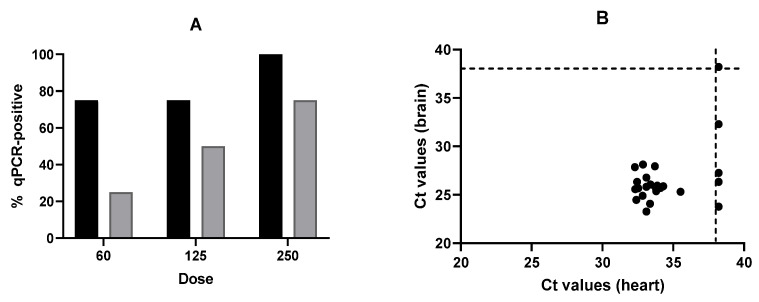
(**A**) Influence of *T. gondii* oocyst dose on the percentage of infected mice determined by qPCR of heart tissue samples. Control samples (black bars) (untreated) and PEF-treated samples (grey bars) (15 kV/cm, 50 kJ/kg, 225 µs). (**B**) Correlation between qPCR Ct values obtained in heart and brain tissue for mice inoculated with oocysts of *T. gondii.* The dashed line indicates the detection limit of the assays. Error bars correspond to the standard deviation.

Results are shown in Figure 2A, which shows a trend similar to that reported for brain qPCR regarding the influence of the dose and the effect of PEF on *T. gondii* oocysts. Higher doses resulted in larger infectivity percentages, and the PEF treatment reduced them. However, comparison of the results obtained from heart and brain tissues (Table 2) indicated that 21% of the mice classified as positive by qPCR analysis of brain tissue were negative when heart tissue was analyzed. Furthermore, no significant correlation (Pearson: r = −0.098, *p* = 0.6908; Spearman: r = −0.066, *p* = 0.7889) was found between the Ct values obtained for the two tissues (Figure 2B). Altogether, these results support the conclusions drawn above regarding the effect of the dose and the impact of PEF on oocyst viability/infectivity, indicating that qPCR analysis of heart tissue would be less sensitive than qPCR of brain tissue for the detection of *T. gondii* infection in mice, at least under the conditions assayed in this study.

##### Indirect Immunofluorescence Assay (IFA)

**Figure 3:**
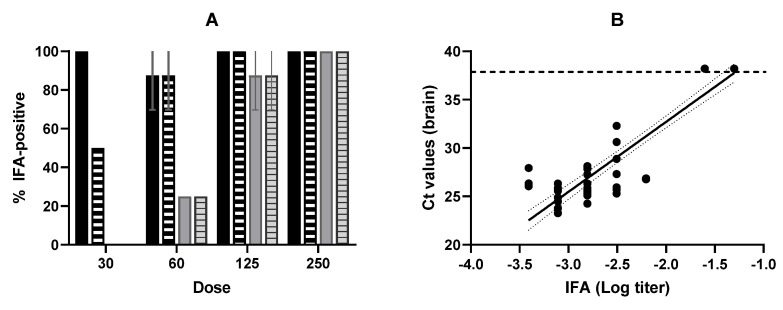
(**A**) Influence of *T. gondii* oocyst dose on the percentage of infected mice determined by IFA. Control samples (black bars) (untreated) and PEF-treated samples (grey bars) (15 kV/cm, 50 kJ/kg, 225 µs). Filled bars: Positive = titer ≥ 1:20; Striped bars: Positive = titer ≥ 1:80. (**B**) Correlation between brain qPCR Ct values and logarithm of IFA titers in mice infected after oral inoculation with *T. gondii* oocysts. The thin dashed line indicates the detection limit of the assays, and the thin lines indicate the 95% deviation of the linear regression line. Error bars correspond to the standard deviation.

The percentage of mice infected after the oral administration of varying doses of PEF-treated and non-treated (control) oocysts, as determined by IFA, is shown in Figure 3A, which indicates that PEF treatments reduced the viability/infectivity of *T. gondii* oocysts. For instance, when inoculated with 30 oocysts, all the mice exposed to control oocysts were IFA-positive, whereas only 50% were positive for PEF-treated oocysts. As for qPCR, all negative controls tested negative using IFA. By contrast, when mouse infection was evaluated by means of IFA, the effect of the dose on the number of infected mice was not as evident, at least when mice were inoculated with non-treated (control) oocysts.

The correspondence between IFA and qPCR (brain) results was also examined (Table 3 and Figure 2B). Results obtained with the two techniques were very similar, as can be observed in Table 3. All mice that were classified as positive (infected) by qPCR were also classified as positive by IFA. Similarly, all mice classifying as negative (uninfected) by IFA were also classified as negative by qPCR. Thus, the difference between the two techniques was due to the fact that a certain proportion of mice (5%) were classified as infected by IFA but as non-infected by qPCR. However, a perfect correlation between the results obtained by qPCR of brain tissue and by IFA would have been found if, instead of using the initial/standard criteria (Positive = titer ≥ 1:20), we had used a slightly higher titer to define positivity/infection (Positive = titer ≥ 1:80).

The good correlation between the two techniques was not limited to how they classified (infected vs. uninfected) mice (Table 3); we also found a strongly significant correlation between the Ct values (qPCR) and the titer (IFA) (Pearson: r = 0.9510, *p* < 0.0001; Spearman: r = −0.8184, *p* = 0.0001) (Figure 3B). In fact, a linear relationship between the Log of the titer and the Ct values was observed, with an R^2^ value of 0.884 and a root mean square error (RMSE) of 1.986 (Figure 3B). This strong correlation suggests that IFA could serve as a reliable preliminary indicator of *T. gondii* infection.

#### 3.1.2. Cell Culture Assay

**Figure 4:**
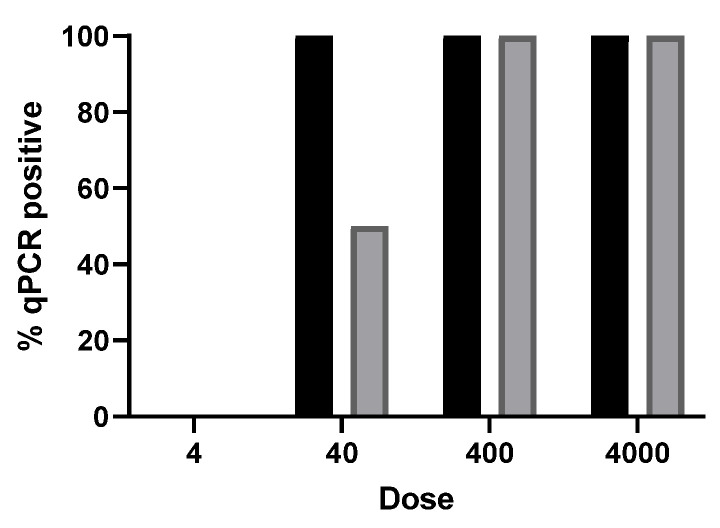
Influence of *T. gondii* oocyst dose on the percentage of wells with infected fibroblasts (determined by qPCR after 7 days of culture). Control samples (black bars) (untreated) and PEF-treated samples (grey bars) (15 kV/cm; 50 kJ/kg; 225 μs). Error bars correspond to the standard deviation.

Figure 4 shows the influence of *T. gondii* oocyst dose on the percentage of wells with infected fibroblasts of control and PEF-treated oocysts determined by qPCR after 7 days of incubation. Wells with control oocysts were consistently positive across all doses tested, except the four-oocyst dose, confirming high infectivity of the oocysts under standard conditions. In contrast, PEF-treated oocysts exhibited a reduction in the number of positive wells for the inoculation dose of 40 oocysts. These in vitro results align well with the in vivo findings described above. Thus, in the mouse experiments, significant differences in infection rates were observed between PEF-treated and control groups at doses of 30 and 60 oocysts. These results obtained in cell cultures support the conclusion that PEF treatment reduces oocyst infectivity.

### 3.2. Effect of PEF on T. gondii Bradyzoites

**Figure 5:**
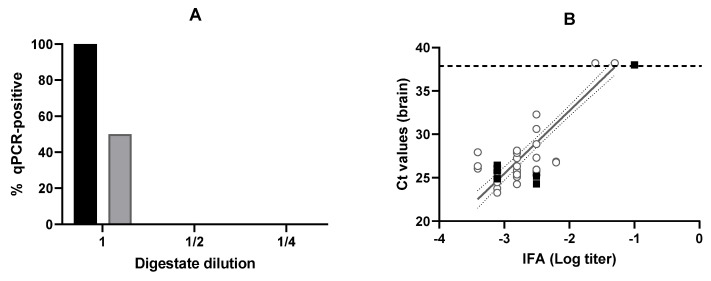
(**A**) Influence of digestate dilution of *Toxoplasma gondii*-infected meat on the percentage of infected mice determined by qPCR of brain tissue samples. Control (untreated) samples (black bars) and PEF-treated samples (grey bars) (3.3 kV/cm; 27 kJ/kg; 1600 µs). (**B**) Correlation between brain qPCR Ct values and logarithm of IFA titers in mice infected with *T. gondii* oocysts. Grey empty circles (○) correspond to the values obtained for *T. gondii* oocysts, and the thin lines indicate the 95% deviation of their linear regression line. Filled black squares (■) correspond to the values obtained for *T. gondii* bradyzoites. The thin dashed line indicates the detection limit of the assays. Error bars correspond to the standard deviation.

To assess the effect of PEF on the survival of *T. gondii* bradyzoites, the digestion extract obtained from PEF-treated (3.3 kV/cm; 27 kJ/kg; 1600 µs) muscle or untreated muscle (control) was inoculated into mice at three doses/dilutions (undiluted, 1/2, and 1/4 in PBS). As shown in Figure 5A, PEF application resulted in a 50% reduction in the number of infected mice following inoculation of the meat digest, as determined by qPCR analysis of brain tissue. In contrast, none of the other dilutions (regardless of whether the meat had been treated or not), nor the negative controls (100 °C; 15 min), resulted in infection in any of the mice. Notably, the same pattern was observed in the cell culture bioassays, corroborating the reduced infectivity of the PEF-treated meat.

The good correspondence between the results obtained by IFA and qPCR analysis of brain tissue was also observed in the case of infection with bradyzoites (digested tissue). Thus, Figure 5B illustrates how the data obtained for bradyzoites are consistent with those obtained for oocysts, despite corresponding to different developmental stages of the parasite and different routes of inoculation. These results corroborate the ability of PEF treatments to inactivate *T. gondii* in different developmental stages and demonstrate a strong concordance between IFA results and brain tissue qPCR analysis.

Finally, all the findings presented above highlight the potential of in vitro cell culture systems as a viable and ethically preferable alternative for the preliminary assessment of *T. gondii* oocyst viability and treatment efficacy, in line with the 3R principle (Replacement, Reduction, and Refinement).

## 4. Discussion

As stated above, this study’s main objective was to evaluate the effect of PEF treatments on the viability of different evolutional stages of *T. gondii*. To address this, cell culture assays and in vivo experiments in mice were conducted to evaluate oocyst infectivity after PEF treatments. Our secondary aim was to compare these experimental approaches and techniques in terms of their effectiveness in assessing *T. gondii* infectivity.

IFA is commonly used as a rapid screening method to detect specific antibody responses in mice. It is also safe, inexpensive, sensitive, and easy to carry out [45]. However, to confirm infection, qPCR analysis is usually performed on a target tissue collected post-mortem (in our case, two months after inoculation), given qPCR’s higher specificity compared to IFA. Moreover, qPCR detection can be performed on several different tissues, including the brain and the heart, which are key target organs for *T. gondii* cyst formation [10,46]. The results reported herein demonstrate an excellent agreement between IFA and qPCR of brain tissue (Table 3, Figure 3B and Figure 5B). It is worth noting that the correlation was considerably stronger than the one observed between the qPCR results of brain and heart tissues (Figure 2B). Furthermore, the slight differences we observed between the two techniques when the lowest titer tested (1:20) was employed to determine infection status in mice after oocyst inoculation disappeared when we used the 1:80 titer instead (Table 2), a result consistent with the statistically significant correlation between IFA titers ≥ 1:80 and qPCR positivity (as defined in Berdejo et al. [47]) in tissue samples previously reported by Herrero et al. [48]. These discrepancies probably reflect that these mice, while positive by IFA, exhibited very low titers, consistent with a weak immune response. This may result from inoculation with a low number of viable oocysts, which is insufficient to establish or sustain an infection, thereby explaining the negative qPCR results. Notably, all such cases occurred in mice receiving the lowest dose (30 oocysts), which is below the reported ID_50_ for untreated oocysts (34.57 as indicated above). This hypothesis would require further confirmation.

The results also indicate that qPCR analysis of heart tissue (Figure 2A) tends to be less sensitive than analysis of brain tissue, i.e., it may lead to false negatives (classification of infected mice as uninfected). This is consistent with previous findings indicating that parasite counts in infected animals of several species (including pigs, sheep, and mice) were usually higher in the brain than in heart tissue [46,49,50]. Furthermore, the differences in Ct values observed in our study are within the range reported by Juránková et al. [48]. This similarity, however, should be interpreted with caution, given the differences in species and methodologies between that study and ours. In addition, it cannot be ruled out that the differences in Ct (i.e., parasite counts) between heart and brain tissues reported here (Figure 2B) might be partially due to the fact that we used the same technique for tissue disruption of both organs, although their structural tissue characteristics differ considerably. Tissue disruption in muscle may be less efficient, potentially leading to an underestimation of the number of parasites. Further research would be required to clarify this point.

Although bioassay is considered the reference method for evaluating the efficacy of treatments against parasitic infections, it presents several limitations [25]: bioassays are expensive, time-consuming, and require the use of live animals, which raises significant ethical concerns, particularly for long-term studies [51]. As highlighted by Rusche [52], the use of in vivo models should be minimized whenever possible in accordance with the 3R principle. In this context, in vitro alternatives such as cell culture have gained interest as complementary tools [53]. However, although these methods offer advantages in terms of cost and ethical acceptability, their application remains primarily confined to experimental settings and specific purposes [51]. As a consequence, our study also aimed to explore the potential correlation between in vivo and in vitro systems, and to evaluate the suitability of cell-based assays as a complementary method for assessing *T. gondii* viability and (PEF) treatment efficacy.

The results obtained with cell cultures aligned with those from the mouse bioassay model in demonstrating the ability of PEF to inactivate *T. gondii* oocysts (Figure 4) and bradyzoites (Figure 5). Even more surprisingly, they suggest that the number of oocysts required to establish infection in mice and in cell culture may fall within a similar range. However, this latter finding would require specifically designed studies for confirmation and should therefore be interpreted with extreme caution, particularly given the marked differences between the two experimental models. In any case, our data clearly suggests that cell culture assays could be useful, at least as a screening tool, for assessing oocyst infectivity and for evaluating the impact of various agents or technologies on their viability and infectivity. In this context, this technique could potentially replace the mouse bioassay, which is time-consuming, costly, unsuitable for routine diagnostic use, and requires the use of animals.

Regarding our study’s primary objective, this is, to the best of our knowledge, the first report to examine the effect of PEF on the viability and infectivity of *T. gondii*, providing evidence of this technology’s capability to inactivate them, consistently demonstrated across all the tested techniques to evaluate its viability in this work. Moreover, the obtained results demonstrate that this technology is capable of inactivating this parasite in different developmental stages (oocysts and bradyzoites, Figure 1A, Figure 2A and Figure 5A). It should also be noted that the treatment conditions were carefully selected to limit the energy input (50 kJ/kg in aqueous solution and 27 kJ/kg in meat), thereby ensuring that parasite inactivation occurred under non-thermal conditions, minimizing the impact of the process on the characteristics of the treated matrix—particularly in the case of meat—and favouring both process scalability and the economic feasibility of industrial implementation. In any case, given that oocysts represent highly resistant stages and that no sensory properties need to be preserved in aqueous solutions, the intensity of the applied treatment (both electric field strength and energy input) was substantially higher, whereas milder treatments were applied to meat due to its greater sensitivity.

The capability of PEF to inactivate oocysts is somewhat unexpected, as oocysts represent a resistant stage of *T. gondii*, where the parasite (in its sporozoite form) is encased within a complex, multilayered structure consisting of two protein-rich walls: the oocyst and sporocyst walls [5,6,54]. In comparison, other resistant biological forms, such as bacterial spores, are typically considered or have been shown to be resistant to PEF [27]. However, certain ascospores and conidiospores of yeast and moulds have proven sensitive to PEF [55]. Recent studies have also shown that certain bacterial spores can be inactivated through combined processes, including PEF, although thermal effects affected the spores’ sensitivity to PEF [56,57,58]. As previously indicated, the combined effects of PEF and heat were not expected, as sample temperatures remained below 35 °C. Further studies would be required to determine the causes of this particular, rather unexpected effect of PEF on *T. gondii* oocysts. Although all forms of microbial resistance share certain analogies, they also display substantial differences that can account for their distinct responses to PEF.

In the case of bradyzoite inactivation, direct comparisons with other microorganisms are more difficult to establish. Thus, it is well known that electroporation is strongly dependent on cell size [59]. Considering the dimensions of bradyzoites (approximately 1.5 × 7 µm), the observation that their viability is partially affected by a treatment of 3.3 kV/cm is consistent with the estimations reported by these authors, at least under certain scenarios. However, bradyzoites are enclosed within tissue cysts. Although this designation might initially suggest a protective role, the obtained results in this work do not support this assumption.

Finally, our results, based on the applied PEF treatments, indicate that the level of inactivation was around 50 in both cases. This suggests that PEF treatment may represent a promising non-thermal approach for significantly reducing the infective potential of environmental oocyst contamination in water-based systems and of tissue cysts in meat. Although this level of inactivation is still far from what would be required to ensure food safety, further research could pinpoint combined processes (i.e., PEF combined with sublethal or lethal temperatures, antimicrobials, etc.) and/or explore other treatment conditions (e.g., higher field strengths and/or energy inputs) that would precisely demonstrate this technology’s potential for *T. gondii* inactivation. Considering the environmental persistence of *T. gondii* oocysts in water and plant-derived foods [12], PEF technology could find practical application as a non-thermal disinfection method in such matrices. Its incorporation into water treatment systems or into the processing of fresh produce and fruit juices could help reduce the risk of foodborne transmission, offering a promising alternative to conventional chemical or thermal treatments. Similarly, given the high prevalence of *T. gondii* not only in pigs but also in other food-producing species such as broiler chickens, sheep, and cattle, contaminated meat represents an equally important, if not greater, route of human exposure [43,47,60]. In this context, the incorporation of PEF into meat processing could also help reduce the risk of transmission through this route, offering a valuable complement to existing food safety measures. This is particularly relevant because the parasite is not routinely targeted by conventional meat inspection, allowing tissue cysts to persist in meat destined for human consumption.

## 5. Conclusions

This study demonstrated that treatment with Pulsed Electric Fields (PEFs) significantly reduced the infectivity of *T. gondii* oocysts (15 kV/cm for 225 µs and 50 kJ/kg) and bradyzoites (3.3 kV/cm for 1600 µs and 27 kJ/kg) in an aqueous solution and muscular tissue, respectively. The treatment’s efficacy was evidenced both in vivo, through serology and qPCR in mouse tissues, and in vitro, using cell cultures. In all cases, a marked decrease in the infectivity rate of PEF-treated *T. gondii* was observed, suggesting an inactivation effect of PEF on the parasite’s viability. Our results support the potential use of PEF as a non-thermal strategy for inactivating oocysts in water and liquid food products and of tissue cysts (bradyzoites) in meat. In any case, it should be noted that in this study only a single PEF treatment was applied. While this treatment demonstrated an inactivation effect, higher or alternative PEF treatments should be explored to achieve greater inactivation of both oocysts and bradyzoites. Additionally, no food matrix quality parameters were evaluated. More specific future studies should assess the impact of different PEF treatments on both parasite inactivation and quality parameters of various food matrices, optimizing treatment conditions while ensuring food safety and maintaining organoleptic properties. On the other hand, our study highlighted the utility of in vitro assays as complementary tools to bioassays, thereby promoting more ethical and sustainable methods in parasitological research.

## Figures and Tables

**Table 1:** Primers used in this study.

Name	Sequence	Reference
RocFw	TAGACGAGACGACGCTTTCC	[43]
RocRv	TCGCCCTCTTCTCCACTCT	[43]

**Table 2:** Contingency table comparing qPCR results from brain and heart tissues to determine the number of mice infected after oral inoculation with *T. gondii* oocysts. The values indicated represent the percentage of matching results.

		qPCR (Brain)	
		Positive (Infected)	Negative (Uninfected)
**qPCR (heart)**	Positive (Infected)	89%	0%
	Negative (Uninfected)	21%	100%

**Table 3:** Contingency table comparing qPCR results from brain and IFA to determine the number of mice infected after oral inoculation with *T. gondii* oocysts. The values indicated represent the percentage of matching results. Data in parentheses correspond to the comparison using a 1:80 titer instead of 1:20.

		qPCR (Brain)	
		Positive (Infected)	Negative (Uninfected)
**IFA**	Positive (Infected)	95 (100)%	5 (0)%
	Negative (Uninfected)	0 (0)%	100 (100)%

## Data Availability

The original contributions presented in this study are included in the article. Further inquiries can be directed to the corresponding author.

## Abbreviations

The following abbreviations are used in this manuscript:

Ct	Cycle threshold
qPCR	Quantitative polymerase chain reaction
IFA	Indirect immunofluorescence assay
ID_50_	50% infectious dose
PBS	Phosphate-Buffered Saline
DMEM	Dulbecco’s Modified Eagle’s Medium
HHP	High-hydrostatic-pressure processing
PEF	Pulsed electric field
